# The double-plate fixation technique prevents varus collapse in AO type C3 supra-intercondylar fracture of the distal femur

**DOI:** 10.1007/s00402-023-04953-4

**Published:** 2023-06-22

**Authors:** Chang-Heng Liu, Ping-Jui Tsai, I-Jung Chen, Yi-Hsun Yu, Ying-Chao Chou, Yung-Heng Hsu

**Affiliations:** 1 Department of Orthopaedic Surgery, Chang Gung Memorial Hospital, Linkou Medical Center, No. 5, Fusing St., Gueishan Dist., Taoyuan City, 333423 Taiwan (ROC); 2 Bone and Joint Research Center, Chang Gung Memorial Hospital, Linkou Medical Center, Taoyuan City, Taiwan (ROC)

**Keywords:** Distal femur fractures, Supra-intercondylar femoral fractures, Lateral locking plate, Double plate, Varus collapse, Varus malunion

## Abstract

**Introduction:**

Varus collapse followed by osteosynthesis for distal femoral fractures with conventional implants has been well documented but is seldom mentioned in fractures managed with locking plates. The purpose of this study was to assess the incidence of varus collapse after treating complex supra-intercondylar fractures of the distal femur (AO type C3) using a Single Plate (SP) or Double Plate (DP) fixation technique.

**Materials and methods:**

We retrospectively reviewed 357 patients with distal femoral fractures who were treated at our hospital between 2006 and 2017. After excluding cases of infection, malignancy, periprosthetic fracture, revision surgery, pediatric fracture, and extra-articular fracture, 54 patients were included in the study. All demographic data and radiological and clinical outcomes were reviewed and analyzed.

**Results:**

There were 54 patients enrolled into this study with age from 15 to 85 years old (mean 41.6, SD = 19.9), and 32 of them were open fractures (59%). The patients were further divided into either an SP (*n* = 15) or a DP group (*n* = 39). Demographics, including age, sex, injury severity score, and open fracture type, were all compatible between the two groups. The overall nonunion rate was 25.9% (*n* = 14; 6 from the SP and 8 from the DP group; *p* = 0.175). The varus collapse rate was 9.3% (*n* = 5; 4 from the SP and 1 from the DP group (*p* = 0.018).

**Conclusions:**

The varus collapse rate after osteosynthesis with a single lateral locking plate could be as high as 26.7% in AO type C3 fractures of the distal femur, which would be decreased to 2.6% by adding a medial buttress plate. Surgeons should consider DP fixation to avoid varus collapse in severely comminuted complete intra-articular fractures of the distal femur.

## Introduction

Distal femoral fractures account for 3–6% of all femoral fractures, with a bimodal age distribution [1, 2]. In young patients, fractures are usually caused by high-energy trauma, such as motor vehicle accidents or falls. In the elderly, even low-energy trauma can cause distal femoral fractures, such as slipping from a standing level. Fracture patterns are also different for each type of trauma mechanism. In high-energy trauma, supracondylar fractures are more likely to have intercondylar extensions, and the metaphyseal region is usually comminuted. Isolated supracondylar fractures are more common in patients with low-energy trauma. Regardless of the type of injury or severity of fracture, the deforming force of the femur always turns the proximal fragment into a flexion deformity (pulled by the hamstring muscle) and the distal part into an adduction and extension deformity (pulled by the adductor and gastrocnemius muscles)[3]. The injured limb is also shortened.

Surgical treatment is the mainstay therapy of most distal femoral fractures, except in non-displaced or minimally displaced fractures, or in severely ill patients with very high anesthesia and surgery risk. Conservative treatments include protected or non-weight-bearing activity in a hinged knee brace until the callus is present or bridging callus across the fracture side is observed by radiography. Decreased range of motion of the knee joint, wasting of limb muscle, pressure ulcer over the dependent part, venous thromboembolism, and pulmonary distress are possible complications associated with conservative treatments [4–6].

There are many surgical methods, and the choice of implants depends on the fracture classification. For extra-articular fractures known as AO type 33-A, either an intramedullary nail or a plate could be the option [7]. The success rate of the procedure was satisfactory. For partial-articular fractures (AO type 33-B), namely, condylar fractures involving the coronal or sagittal plane of the distal femur, fixation with interfragmental screws is usually sufficient [8]. An additional buttress plate provides more stability under certain circumstances. For complete intra-articular fractures (AO type 33-C3), anatomical reduction of the articular surface and restoration of leg length and alignment are major challenges for orthopedic surgeons. Conventional implants, such as condylar plates or dynamic condylar screws, lead to unsatisfactory outcomes due to implant design and poor fixation stability over distal fragments, especially in cases of severe articular comminution and huge metaphyseal bone defects [9, 10]. The locking plate system plays an important role in the treatment of complex supra-intercondylar fractures of the distal femur [8, 11–13]. Adequate stability, preservation of the soft tissue envelope, and early mobilization are the keys to success [14, 15].

By treating complex supra-intercondylar fractures of the distal femur with a Condylar Buttress Plate (CBP), the varus collapse rate could be as high as 42% [9]. It indicates that lateral side-based implants are inadequate, especially for complex fracture patterns. The lack of medial buttress in the extremely comminuted metaphysis is believed to be the reason for the failure. The occurrence of varus collapse in patients treated with locking plates remains undetermined and seldom mentioned in the literature. The aim of this study was to assess the incidence of varus collapse after treating complex supra-intercondylar fractures of the distal femur with a Single Plate (SP) or Double Plate (DP). We hypothesized that the varus collapse rate could be reduced by the DP fixation technique compared with the SP fixation method.

## Materials and methods

### Inclusion and exclusion criteria

A total of 357 patients who were diagnosed with distal femoral fractures and underwent osteosynthesis surgery with the distal femoral locking compression plate (less invasive stabilization system for LISS and DF LCP, Synthes, Paoli, PA, U.S.A.) between 2006 and 2017 in our hospital were included in this series. We excluded patients with periprosthetic fractures around the knee joint (*n* = 92), extra-articular or partial-articular fractures (*n* = 65), delayed surgery for more than 4 weeks between injury and definite treatment (*n* = 3), revision surgery for femoral shaft malunion or nonunion (*n* = 19), tumor invasion-related pathological fractures (*n* = 5), age under 18 years (*n* = 1), and infection (*n* = 1). Patients who were lost within the first postoperative 12 months (*n* = 59) were also excluded. The remaining 112 complete intra-articular fracture cases were further examined, and only AO type 33-C3 fracture patients were retained. Demographic and clinical data were collected during hospitalization and postoperative 6 weeks, 3, 6, and 12 months. The follow-up was then performed every 6 months until the fracture healed. Early and late postoperative complications such as surgical site infection, wound dehiscence, knee stiffness, implant failure, malunion, and nonunion were also recorded.

The Lateral Distal Femoral Angle (LDFA) was measured as the angle between the axis of the femoral shaft and the joint line of the distal femur [16]. Varus collapse was defined as an increase in LDFA of > 5° measured with plain film compared with the first postoperative plain film. Successful fracture union was defined as three or more continuous bridging bony cortices observed on anteroposterior and lateral radiography, and patients could tolerate full weight-bearing activity without pain [17]. Nonunion was defined as insufficient callus formation or a gap between the fracture site and the discontinuity of the bony cortex on the plain film with cessation of callus progress for at least 3 months or lack of healing by 9 months since injury [18, 19]. Both implant failures and cases that underwent revision surgery for bone grafting were classified as fracture nonunion. Surgical site infection was defined as an inflammatory reaction involving local soft tissue with increased inflammatory markers such as C-reactive protein and erythrocyte sedimentation rate. Superficial infection was defined as inflammation above the fascia level, in contrast to deep infection, in which the bony structure was also involved. All radiographic, clinical, and demographic data were reviewed by three experienced orthopedic trauma surgeons. Additional conferences were arranged for cases with discrepancies until final agreements were reached between all surgeons.

### Surgical techniques

Upon presentation to the emergency room, an advanced trauma life support protocol was carried out for the primary survey and management of trauma patients. Early total care or damage control orthopedics depends on the patient’s general condition and the soft tissue envelope around the injured limb. When a patient has multiple fractures, polytrauma, open fracture, or poor soft tissue condition or is hemodynamically unstable, spanning external knee fixators were used to prevent a second hit injury. For critical patients, such as those in shock or blunt abdominal trauma with internal organ injury who need to undergo laparotomy, orthopedic surgery would be postponed until reaching a relatively stable general condition.

**Fig. 1:**
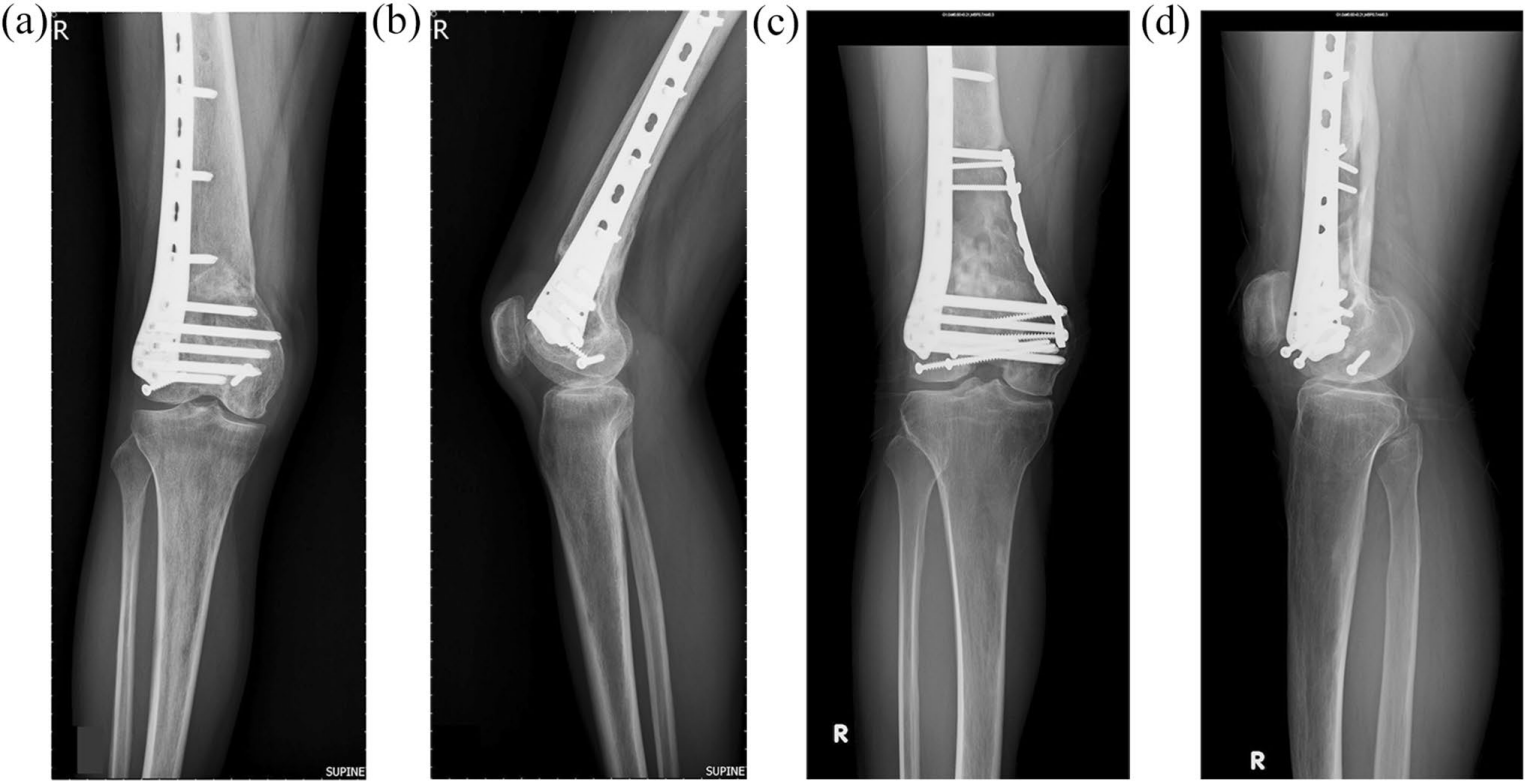
The configurations of plates and screws after osteosynthesis with single lateral locking plate (**a** and **b**) or double plates (**c** and **d**). Note the multiple interfragmental screws were applied in the beginning of the surgery for the reduction and fixation of articular surface. The medial buttress plate was bended to match the curvature of the distal femoral condyle

**Fig. 2:**
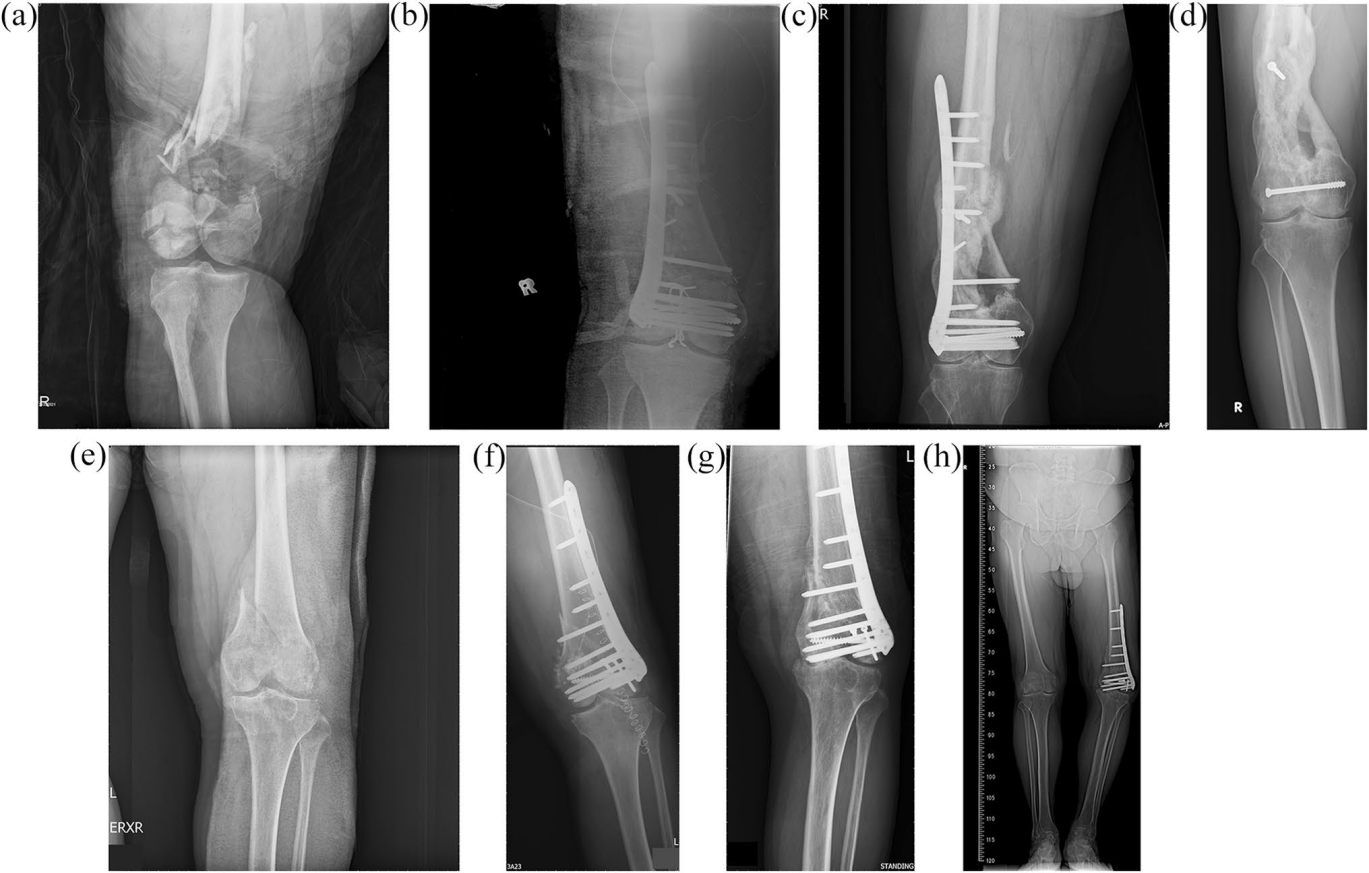
The demonstration of varus collapse with malunion after osteosynthesis with lateral locking plate. (**a** and **b**) Pre and post-operative anteroposterior plain film of an AO type C3, open type IIIa distal femoral and patellar fracture in a 27-year-old male. **c** Varus collapse with loosed screws over the femoral shaft at 27 months after the operation. **d** Malunion with varus deformity at 90 months. The change of LDFA was 11˚. **e** and **f** Pre and post-operative anteroposterior plain film of a closed AO type C3 left distal femoral fracture in a 50-year-old male. **g** and **h** Varus collapse of the medial femoral condyle with malunion at 14 months after the operation. The change of LDFA was 5˚

**Fig. 3:**
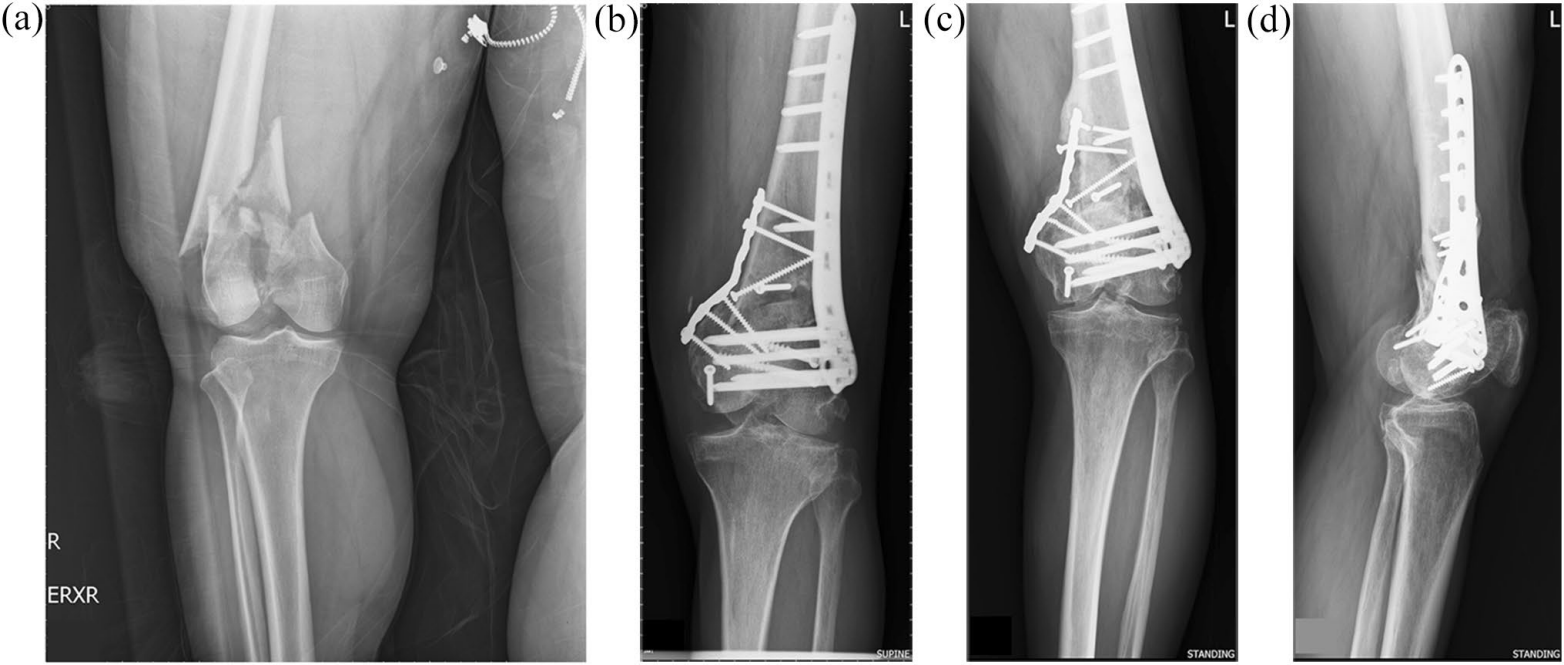
**a** A 59-year-old male with closed AO type C3 left distal femoral fracture. **b** Post-operative anteroposterior plain film after osteosynthesis with double plates. **c** and **d** The fracture was collapsed and the medial buttress plate was loosed at 21 months. The change of LDFA was 5˚

Definite fracture osteosynthesis was performed under general anesthesia. Bumps were placed under the popliteal region to relax tension in the gastrocnemius muscle. The lateral parapatellar approach was used to reduce the articular surface with multiple interfragmentary screws. After anatomically reducing the articular surface, metaphyseal and diaphyseal fractures were reduced and temporarily fixed. There are many tools and methods for limb traction and maintaining reduction, such as Schanz pins, Knowles pins, distractors, temporary spanning external fixators, and manual traction. Surgeons must use the contralateral leg for the leg length and limb axis reference. A distal femoral locking plate (LISS, Synthes) was then applied on the lateral side of the femur via limited open reduction technique, that is, the articular surface was reduced by open approach, and the meta-diaphyseal region and proximal screws insertion were managed by minimally invasive technique. Whether or not to fill the metaphyseal bone defect was dependent on the surgeon’s experience, as well as the bone quality and fracture patterns. An additional buttress plate on the medial side was applied according to the surgeon’s preference (Figs. 1, 2, 3). Closed drainage was left in the knee joint for hematoma evacuation. The wound was then closed layer-by-layer. Neither splinting nor knee bracing was performed after surgery.

### Post-operative rehabilitation

All patients received continuous passive machine training for knee joint range of motion (ROM) right on the day after surgery. Early ROM is essential to prevent knee stiffness and joint contracture. Drainage was removed if the daily amount was lower than 150 ml. Once the hemoglobin level was lower than 10 mg/dL or dropped to 3 mg/dL or less with clinical symptoms, a blood transfusion with red blood cells and plasma was arranged. A rehabilitation program was then initiated. The patients were subjected to physical training for ankle pumping exercises, active knee ROM training, and non-weight-bearing activity with a walker or crutches. Partial-weight-bearing activity was allowed once a callus was present on the radiograph. If a bridging cortex was observed on a plain film, the patients could begin full weight-bearing activity.

### Statistical analysis

To evaluate differences between SP and DP group in categorical variables like gender, injured limb, degree of open fracture, and the complications, Chi-squared analysis was used. For comparison between groups in continuous data like age, ISS score, days from injury to fixation, the screws and holes of implants, time of follow-up and time to union, Student’s t test was used. Statistical significance was defined as *p* value ≤ 0.05.

## Results

Of the 112 acute distal femoral fracture patients, 54 patients belonged to the AO type C3 fracture, and 32 of them were open fracture (59%). All 54 patients were further divided into either SP or DP groups. There were 15 patients in the SP group (7 males, 8 females) with a mean age of 35.4 years, and 39 patients (29 males, 10 females) in the DP group with a mean age of 44.4 years. There was no statistically significant difference between the two groups in the sex ratio, type of open fracture, and Injury Severity Score (ISS) (Table 1).

**Table 1:** The demographics of SP and DP groups

	SP	DP	Total	*p* value
Numbers	15	39	54	N/A
Age	35.4	44	41.6	0.159
*Gender*				0.105
Male	7	29	36	
Female	8	10	18	0.393
Right side	10	21	31	
Left side	5	18	23	
Closed fracture	4	18	22	0.215
Open fracture				0.215
Type I	0	2	2	
Type II	5	13	18	
Type IIIa	5	5	10	
Type IIIb	0	1	1	
Type IIIc	1	0	1	
Spanning external fixators before definite fixation	12	25	37	0.260
From injury to fixation (days)	8.533	8.154	8.259	0.809
Injury severity score (ISS)	14.5	12.2	12.8	0.27

Regarding the implant configurations for fracture fixation in all patients, a mean of 3.5 interfragmental screws were used for intercondylar fixation (3 in the SP group and 3.69 in the DP group, *p* = 0.258). The length of the Synthes LISS locking plates was 8.5 holes (range 7–13) and 9.3 holes (range 5–13) in the SP and DP groups, respectively. The mean length of the medial buttress plate was 8.1 holes in the DP (range 6–10) group. The patients were followed up for a mean of 41.8 months after the operation (47.9 months in the SP group and 39.4 months in the DP group, *p* = 0.182). The mean time to bony union was 18.7 months (22.5 months in the SP group and 17.2 months in the DP group, *p* = 0.19) (Table 2).

**Table 2:** The details of plates and screws configuration in SP and DP groups

	SP	DP	Total	*p* value
Interfragmental screws for intercondylar fracture	3 (0–8)	3.69 (0–8)	3.5 (0–8)	0.258
Holes of locking plate	8.5 (7–13)	9.3 (5–13)	9.0 (5–13)	0.131
Numbers of distal screw of locking plate	6.3 (5–7)	5.9 (5–7)	6.1 (4–7)	0.119
Numbers of proximal screw of locking plate	4.7 (4–6)	4.9 (3–7)	4.8 (3–7)	0.344
Holes of medial buttress plate	N/A	8.1 (6–10)	8.1 (6–10)	N/A
Numbers of distal screw of medial buttress plate	N/A	2.1 (1–4)	2.1 (1–4)	N/A
Numbers of proximal screw of medial buttress plate	N/A	2.2 (1–4)	2.2 (1–4)	N/A

There were three plate malpositions, one wound dehiscence, one superficial surgical site infection, seven joint stiffnesses, and one avascular necrosis of the distal femur in this series. There were 14 nonunion cases, 6 and 8 in the SP and DP groups, respectively, and the overall nonunion rate was 25.9%. There were three malunion patients who had bony union but with varus deformity (two in the SP group, one in the DP group, *p* = 0.183). In the 14 nonunion cases, 6 patients had hardware failure (1 in the SP group and 5 in the DP group, *p* = 0.666), and 2 patients in the SP group had varus collapse of the fracture site (*p* = 0.073). The overall varus collapse rate, including varus collapse with nonunion and malunion, was 9.3% (five patients, four in SP and one in DP, *p* = 0.018) (Table 3).

**Table 3:** The follow-up period, clinical results, and complications of SP and DP groups

	SP	DP	Total	*p* value
Follow-up period (months)	47.9 (24–117)	39.4 (13–100)	41.8 (13–117)	0.182
Union time (months)	22.5 (6–69)	17.2 (13–100)	18.7 (4–69)	0.190
*Early complications*				
Plate malposition	2 (13.3%)	1 (2.6%)	3 (5.6%)	0.183
Wound dehiscence	0	1 (2.6%)	1 (1.9%)	1
Superficial infection	0	1 (2.6%)	1 (1.9%)	1
Deep infection	0	0	0	N/A
*Late complications*				
Overall nonunion	6 (40%)	8 (20%)	14 (25.9%)	0.175
Nonunion with hardware failure	1 (6.7%)	5 (12.8%)	6 (11.1%)	0.666
Varus collapse with nonunion	2 (13.3%)	0	2 (3.7)	0.073
Varus collapse with malunion	2 (13.3%)	1 (2.6%)	3 (5.6%)	0.183
Overall varus collapse	4 (26.7%)	1 (2.6%)	5 (9.3%)	0.018*
Joint stiff	0	7 (17.9%)	7 (13%)	0.171
Avascular necrosis	1 (6.7%)	0	1 (1.9%)	0.278

^*^statistically significant

All nonunion cases underwent revision osteosynthesis surgery. Among the 14 cases, 2 underwent revision osteosynthesis with dynamic condylar screw plus medial buttress plate and 4 with Synthes lateral locking plate plus medial buttress plate and autologous bone grafting at a mean of 16 months after the first operation (range 9–23 months). Bony union was achieved at a mean of 6 months after revision surgery (range 3–16 months). For the other eight nonunion cases, autologous bone grafting was performed at a mean of 11 months after the osteosynthesis surgery (range 6–18 months), and bony union was achieved at a mean of 12 months after the operation (range 6–18 months). In the three varus malunion patients, the mean LDFA change compared to the immediately postoperative change was 7° (11°, 5°, and 5° in each patient) (Figs. 2 and 3).

## Discussion

Osteosynthesis surgery for distal femoral supra-intercondylar fractures is a major challenge for orthopedic surgeons. In AO type C3 distal femoral fractures, the articular surface is broken, and the metaphysis is always comminuted with inadequate bony contact. Post-operative varus collapse with implant failure or varus malunion is common. However, it has seldom been mentioned in the literature. Davison et al. reported that a nonfixed-angled lateral CBP resulted in ˃5° of varus collapse in 42% of comminuted distal femoral fractures [9]. Weng et al. reported a 16.7% varus malunion rate in 34 AO type C fracture patients treated with CBP but 0% in AO type A fractures [20, 21].

In traditional implants, which are known as nonfixed-angle devices, the screws over the proximal or distal fragments are often loosened if there is varus collapse of the fracture site. In comparison, fixed-angle lateral locking plates provide superior stability, especially in the fixation of the distal fragment, which leads to promising clinical results [8, 22]. However, varus deformity still occurs, and its incidence is reported to be 8% in AO type C3 fractures[23].Clinically, any degree of varus malunion resulted in post-traumatic osteoarthritis of the knee joint during the long-term follow-up [24, 25]. A malalignment of < 5° in the coronal plane resulted in better functional outcomes [26].

The DP fixation technique for distal femoral fracture has been discussed in recent years. In the early 1990s, Sanders et al. treated complex distal femoral fractures with the DP fixation technique (both lateral and medial buttress plates) to reduce varus angulation, which resulted in bony union in all nine patients with good functional outcomes [27]. DP provided better fixation stability over a SP, especially in severely comminuted fractures, or in osteoporotic distal femoral intra-articular fractures [28, 29]. Comparing with lateral plate alone, there were great axial and rotational stiffness with less displacement and implants failure in double plating constructs. Thus, for those severe comminuted metaphyseal fractures with risk of instability, varus collapse, or nonunion, additional fixation of medial plate may be considered as a useful mechanical solution [30–33]. Applying a medial plate over the distal half of the anteromedial aspect of the femur is quite safe, and the branches of the superficial or deep femoral artery would not be disrupted even while using the minimally invasive technique [34–36]. There were no vascular injury complications in either the SP or DP groups in this study.

In our series, there were three varus deformity cases with bony malunion: two in the SP (13.3%) and one in the DP (2.6%) group. Under the natural varus stress of the distal femur, the medial femoral condyle is predisposed to loss of screw purchase and then collapses if only a lateral-based locking plate is used for fracture fixation. Under these circumstances, an additional medial buttress plate plays an important role in maintaining coronal alignment and improving varus deformity in AO type C3 fractures. Among the varus malunion cases, a collapsed medial femoral condyle was observed in all three patients. There was one case of complication combined loosened screws over the femoral shaft, which was quite uncommon and seldom mentioned in the literature. In addition, there were another two cases of varus collapse with nonunion in the SP (13.3%) group but none in the DP group. If we consider both varus collapse with malunion and nonunion cases together, the varus collapse rate was 26.7% in the SP and 2.6% in the DP groups, which was significantly different. This result could be interpreted as the configuration of DP offering better stability, especially over the medial metaphyseal region, as well as reducing varus collapse in AO type C3 distal femoral fractures, compared to a single lateral locking plate design. Medial cortical defects in distal femoral fractures were found to be at risk of nonunion; therefore, biological or mechanical support is believed to play a major role in countering the varus deforming force in distal femoral fractures [37]. In one systemic review article, the nonunion rate was 4.5% and the unplanned re-operation rate was 8.5% if treating native distal femoral fractures with medial and lateral dual plating. Since the existing clinical research and results were limited by retrospective study design and small cohort sample size, the encouraging clinical outcomes show the advantages of double plating in those complex intra-articular of highly comminuted extra-articular fractures[38].

Khalil et al. treated 12 AO type C3 closed distal femoral fractures with a lateral locking plate and an additional medial buttress plate. All of the cases achieved radiological union without bone collapse, whereas the surgical approach was very extensile with a V-shaped skin incision, and tibial tuberosity osteotomy was mandatory [39]. Mohamed et al. reported 16 AO type C3 fracture cases treated with double locking plates and routine bone grafting via the medial parapatellar approach. There was no varus collapse, but only one nonunion, and one case needed redo bone grafting [40]. Steinberg et al. collected data from 32 patients with acute fracture, nonunion, and periprosthetic fracture who underwent double locking plate fixation, and 30 cases achieved union. However, there were only three AO type C3 cases in this series [36]. The intraoperative varus stress test could provide a reference for surgeons to determine whether an additional medial buttress plate is needed. However, it is quite objective with individual bias [41]. For extensive metaphyseal comminuted fractures such as AO type A3, C2, and C3 and some very low periprosthetic fractures, the DP fixation technique demonstrates a higher union rate and lower re-operation rate than the single lateral plating technique [42, 43].

In our study, there were six nonunion cases in the SP group and eight in the DP group; the overall nonunion rate was 25.9%. There was no statistically significant difference between the two groups in early or late complications, such as wound infection, hardware failure, joint stiffness, or bony avascular necrosis. The longer surgical duration is another issue under debate [43, 44]. We believe that increasing intraoperative blood loss or surgical time while applying double plates is not a major concern because the medial buttress plate is always applied in the last step. In other words, fracture reduction and fixation of the lateral locking plate are key steps in determining surgical duration or blood loss.

The nonunion rate of distal femoral supra-intercondylar fractures treated with lateral LISS is reported to vary widely, ranging from 9 to 22.1%[45, 46], and open fractures, fracture translation, medial cortical defect, smoking are believed to be a significant risk factor for nonunion[37, 43, 47]. For AO type C3 fractures, the nonunion rate could be as high as 23% [48]. All the cases included in our series were AO type C3 fractures, and 59% of them had open fractures. Incidentally, our definition of nonunion was quite strict: patients who underwent revisional surgery for bone grafting only or redo osteosynthesis even without implant failure were all classified as having bony nonunion. Periosteal destruction and gap defects were common in open fractures which resulted into poor vascularity of the fracture site [49]. With the advent of minimally invasive osteosynthesis technique, the vascularity could be preserved and the complications could be decreased. For the gap defects, additional buttress plate plays an important role as a mechanical support for medial site to counter the varus force [37]. Therefore, the nonunion rate in our study was 25.9%, which is comparable to that reported in the literature.

To our knowledge, this article is currently the largest clinical series comparing single- and double-plate fixation strategies in AO type C3 distal femoral fractures with mid-term to long-term follow-up. We highlighted varus collapse with nonunion and malunion issues, even when the fracture was treated with a lateral locking plate, which has seldom been discussed in the literature. However, the 54 distal femoral fracture surgeries were performed by 11 surgeons with different clinical experience. Whether applying single or double plate for fracture fixation or adding the bone graft were depended on each surgeon’s clinical judgement, which was also biased. Whether the DP fixation technique provides similar benefits to AO type C1 or C2 fractures remains undetermined. Besides, the rate of open fracture in our cohort was too high that was never be seen in the literatures. Since this is a single-centered retrospective study, more case numbers and prospective studies with long-term follow-up are mandatory in the future to exclude possible selection bias.

## Conclusion

Varus collapse followed by osteosynthesis for AO type C3 distal femoral fractures occurs not only in conventional implants such as CBP or dynamic condylar screws but also in lateral locking plate systems. The comminuted metaphyseal region, which lacks the biological or mechanical support to counter the varus deforming force, is believed to be the reason for this. The DP fixation technique could prevent varus collapse with clinical significance compared with the single lateral locking plate fixation method. Thus, surgeons should consider an additional medial buttress plate to prevent varus collapse of the fracture in severely comminuted complete intra-articular fractures of the distal femur.

## Data Availability

Data sharing is not applicable to this article as no datasets were generated or analysed during the current study.
